# Effect of the pressureless post-sintering on the hot isostatic pressed Al_2_O_3_ prepared from the oxidized AlN powder

**DOI:** 10.1038/s41598-022-12456-2

**Published:** 2022-05-17

**Authors:** K. Balázsi, D. Varanasi, Zs. E. Horváth, M. Furkó, F. S. Cinar, C. Balázsi

**Affiliations:** 1 Thin Film Physics Department, Institute of Technical Physics and Materials Science, Centre for Energy Research, Konkoly-Thege M. Str. 29-33, Budapest, 1121 Hungary; 2 Metallurgical and Materials Engineering Department, 34469 Maslak, Istanbul, Turkey

**Keywords:** Ceramics, Design, synthesis and processing

## Abstract

The effect of the pressureless post-sintering in hydrogen on the structural and mechanical properties of the hot isostatic pressed Al_2_O_3_ prepared by oxidized AlN powder has been studied. The micrometer size AlN powder has been oxidized in air at 900° C and sintered by hot isostatic pressing (HIP) at 1700 °C, 20 MPa nitrogen atmosphere for 5 h. Pressureless sintering (PS) has been applied for all HIP sintered samples in H_2_ gas at 1800° C for 10 h. It has been shown that the oxidation caused a core–shell AlN/Al_2_O_3_ structure and the amount of Al_2_O_3_ increased with increasing of the oxidation time of the AlN powder. For the first time, the green samples obtained from oxidized AlN powder have been successfully sintered first by HIP followed by post-sintering by PS under hydrogen without adding any sintering additives. All post-sintered samples exhibited the main α-Al_2_O_3_ phase. Sintering in H_2_ caused the full transformation of AlN to α-Al_2_O_3_ phase and their better densification. Therefore, the hardness values of post-sintered samples have been increased to 17–18 GPa having apparent densities between 3.11 and 3.39 g/cm^3^.

## Introduction

Aluminum nitride (AlN) is an alternative refractory ceramic material being used in various range of applications such as optics, electronics and computer circuits for its unique thermal and electrical properties. It has a really high degree of thermal stability and wear resistance while exhibiting a low density^1^. AlN can be obtained either by carbo-thermal reduction of alumina (Al_2_O_3_) or by nitridization of aluminum (Al)^1,2^. AlN exhibits covalent bonding and generally has been sintered around temperatures higher than 1600 °C under the presence of sintering additives acting as oxygen absorbers^2^. On the other hand, Al_2_O_3_ is a simple covalent oxide of aluminum which is generally formed at the surface of pure aluminum. The growing trend of the key issue of the microstructure of the oxide layer and its effect on the oxidation behavior of AlN ceramics is still unclear^3,4^. Al_2_O_3_ has some known phase allotropes. The most commonly identified phase although other intermediary phases evolve during the oxidation process is the γ-Al_2_O_3_^5^. However, these phases are mostly unstable and disintegrate at higher temperatures^5^. These thin aluminum oxide films have been increasingly used in various types of electronic devices as dielectric and tunneling barriers^6^. Zheng et al. fabricated the AlN-Al_2_O_3_ composite ceramic by heat treating Al_4_O_4_C porous ceramic under N_2_ atmosphere above 1500 °C. They showed, that the granular AlN and Al_2_O_3_ particles integrated with each other and closely connected at their grain boundary^7^. Oxidation of AlN ceramics is complicated because of the process is influenced by various factors^8^. Moreover, the oxidation of AlN has been shown to lead to improvements in the adhesion of deposited metal layers in several electronic package applications^8^. Yeh et al. studied the oxidation mechanism of AlN particles through microstructure observation^9^. They confirmed the formation of porous oxide layer on the surface of AlN. The oxidation kinetics was therefore fast and this reaction induced an increased in thickness of oxide layer. The reaction stopped when the pores were no longer interconnected. Korbutowicz et al. studied the oxidation rates of aluminum nitride thin films^10^. They observed the quick diffusion and the oxygen gradient in AlN layers: aluminum nitride inside has been infected with oxygen, due to the surface of aluminum oxide layer revealed a high porosity. The mentioned results are in good agreement with investigations made by Zheng et al.^9^. Maghsoudipour et al. investigated the oxidation behavior of AlN samples in air at elevated temperatures up to 1300 °C gaining different amounts of Al_2_O_3_^11^. The amount of AlN and AlON phases in samples controlled the oxidation behavior of such composites. In samples, having high amount of AlN, the high volume of the evolved nitrogen gas can crack the sample causing further oxidation. Cao et al. also investigated the mechanism of Al_2_O_3_ core formation in AlN films during oxidations^12^. A core–shell structure composed of the AlN core wrapped in the continuous Al_2_O_3_ shell layer has been formed with weak bonding between the core/shell interface and neighboring Al_2_O_3_ shells. The sintering process is more difficult especially for AlN ceramic. The sintering temperature and time must be suitable for each composition (AlN or Al_2_O_3_). On the other hand, the processing method is influencing the obtained microstructure, reduces the grain size and increases the densification of final sintered ceramic. Hot isostatic pressing (HIP) has unique advantages in promoting the compactness of parts, eliminating void defects, reducing segregation and improving the mechanical properties of the ceramics. The presence of more vacancies and pores in oxide core layer can enhance the sintering by offering a higher chance for lattice diffusion^13^. The HIP sintering of Al_2_O_3_ ceramics has a long history of development, therefore is the most familiar for use in the processing of the many existing ceramics materials^14^. Prosvirnin et al. communicated that in the production of oxynitride ceramics micro-additives of sintering components such as Y_2_O_3_, La_2_O_3_, and others are used^15^. The main sintering additive used in oxynitride ceramics is Y_2_O_3_, which has excellent physical and chemical properties, such as high melting point (2430° C) and the density is 5.01 g cm^−3^^15^. Its presence can facilitate the liquid phase during sintering, which is beneficial for compacting and removing pores. Varanasi et al. first oxidized AlN powders for 3, 10 or 20 h and after that sintered by HIP for the first time the dense AlN-Al_2_O_3_ composite without Y_2_O_3_ sintering additives^16^. The sintered samples showed the presence of only α-Al_2_O_3_ besides AlN proving that the sintering results in disintegration of θ-Al_2_O_3_ phase. Their experiments also provided that the densification of sintered ceramics can be achieved by HIP at lower temperatures^16^.

Hydrogen can facilitate the detachment of protective oxide layer from the metals and alloys. The degradation is usually accelerated at elevated temperatures in many industrial applications^17^. Li et al. studied the effect of hydrogen on the integrity of aluminum–oxide interface at elevated temperatures^17^. Anya et al. used the pressureless sintering in hydrogen to obtain Al_2_O_3_-SiC composites^18^. They reported exploration of the effects of sintering variables on the final density and resultant Young’s modulus of composites. Taun et al. prepared the Al_2_O_3_-Ni composites by pressureless sintering in H_2_^19^. The sintering had certain effects on mechanical properties of the composites. The toughness of the composites is enhanced by a crack bridging mechanism or by microcrack toughening. However, the strength of the composites is decreased significantly as the microcracks are formed^19^. Our previous study of the structural and mechanical characterizations of HIP sintered AlN-Al_2_O_3_ was discussed in^16^. A combination of HIP and PS post-sintering is proposed in this paper to obtain high-density bodies with higher hardness. In this work, the effect of pressureless sintering in hydrogen on hot isostatic pressed AlN-Al_2_O_3_ prepared from oxidized AlN powder was studied.

## Materials and experimental

**Figure 1:**
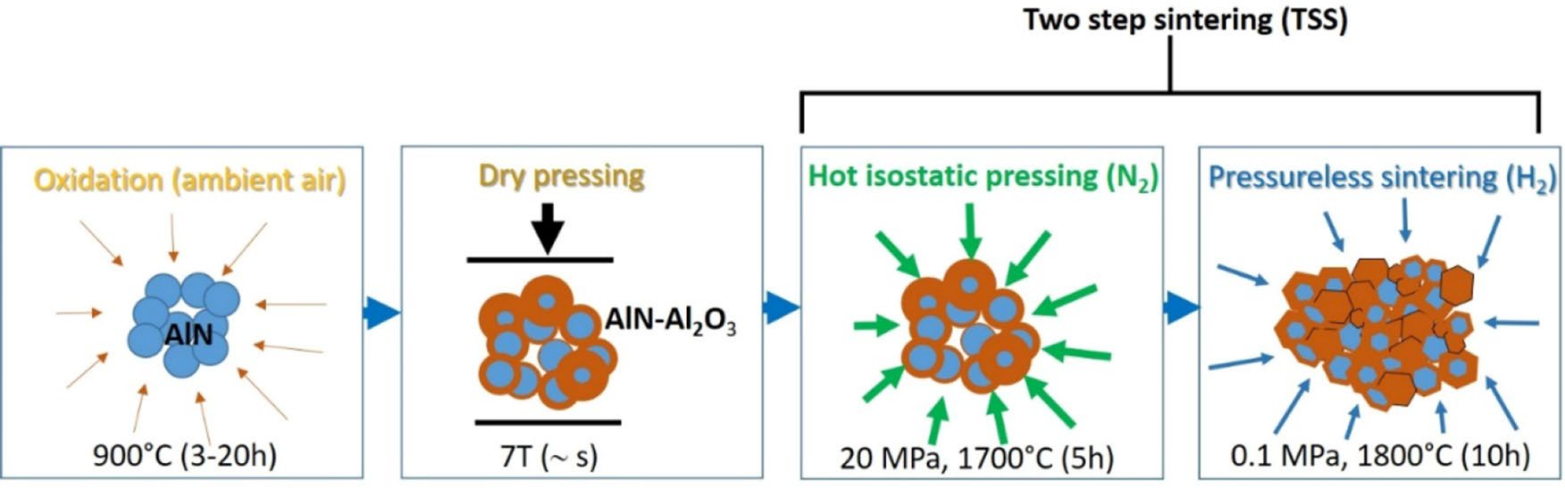
Experimental procedure of Al_2_O_3_ sintered sample preparation from AlN powder.

Base AlN ceramic powders with purity of 98 wt% and the average size of 1.3 ± 0.5 μm (H.C. Starck GMBH, Berlin) have been oxidized in ambient atmosphere at 900 °C for 3, 6, 10 and 20 h respectively. The oxidized AlN powders have been pressed by dry press at 7t. After it, the green bodies have been embedded to BN powder in a graphite crucible and sintered by hot isostatic pressing (HIP, ABRA type) at pressure of 20 MPa, at 1700 °C in an inert gas (N_2_) environment for 5 h. As a post-sintering step, the HIP sintered ceramics have been pressureless sintered (PS) at 1800 °C for 10 h under H_2_ environment simultaneously applying 0.1 MPa pressure. The schematic view of experimental procedure is shown in Fig. 1.

The morphology and the microstructure of the powders and sintered samples have been characterized by scanning electron microscopy (SEM). Leo 1540XH Gemini with lens under SEM-SE mode has been used for the powders and Thermo-scientific Scios 2 for the sintered samples. The surface of the sintered samples have been covered by thin carbon coating to have better resolution and conduction. X-ray diffractometry (XRD) has been carried out using Bruker AXS D8 Discover diffractometer for phase analysis of both the powders and sintered samples. The numbering of the samples after each preparation processes has been indicated in Table 1.

**Table 1:** Identification of samples after each preparation processes.

Oxidation time (h)	Oxidized AlN	HIP sintered AlN-Al_2_O_3_	Pressureless sintered ceramics
0	O_0	HIP_0	PS_0
3	O_3	HIP_3	PS_3
6	O_6	HIP_6	PS_6
10	O_10	HIP_10	PS_10
20	O_20	HIP_20	PS_20

The apparent density of the sintered samples has been measured using Archimedes method where the samples with surface porosity have been immersed in soap water for three days ensuring the complete filling of the pores. The equation used for calculation has been provided in Eq. (1).

1 $$\uprho _{{{\text{apparent}}}} = \frac{{{\text{wt}}\;{\text{of }}\;{\text{dry}}\,{\text{sample}}}}{{{\text{wt}}\;{\text{of }}\;{\text{dry }}\;{\text{sample}} - {\text{wt }}\;{\text{of}}\;{\text{ immersed }}\;{\text{sample}}}} \cdot\uprho _{{{\text{water}}}} $$

The hardness tests of the sintered (HIP and PS) samples have been carried out using Leitz Wetzlar 721464 Vickers microhardness equipment under a load of 19.61 N (2000 P) and the required calculations have been done according to the following equation, Eq. (2),

2 $$ H_{v} = \frac{{1.89 \cdot F \cdot 10^{3} }}{{d^{2} }} $$

where H_v_ is the Vickers hardness, F is the applied force (N) and d is the diagonal length (mm).

## Results and discussion

### Morphological investigations of the powders and the sintered samples

**Figure 2:**
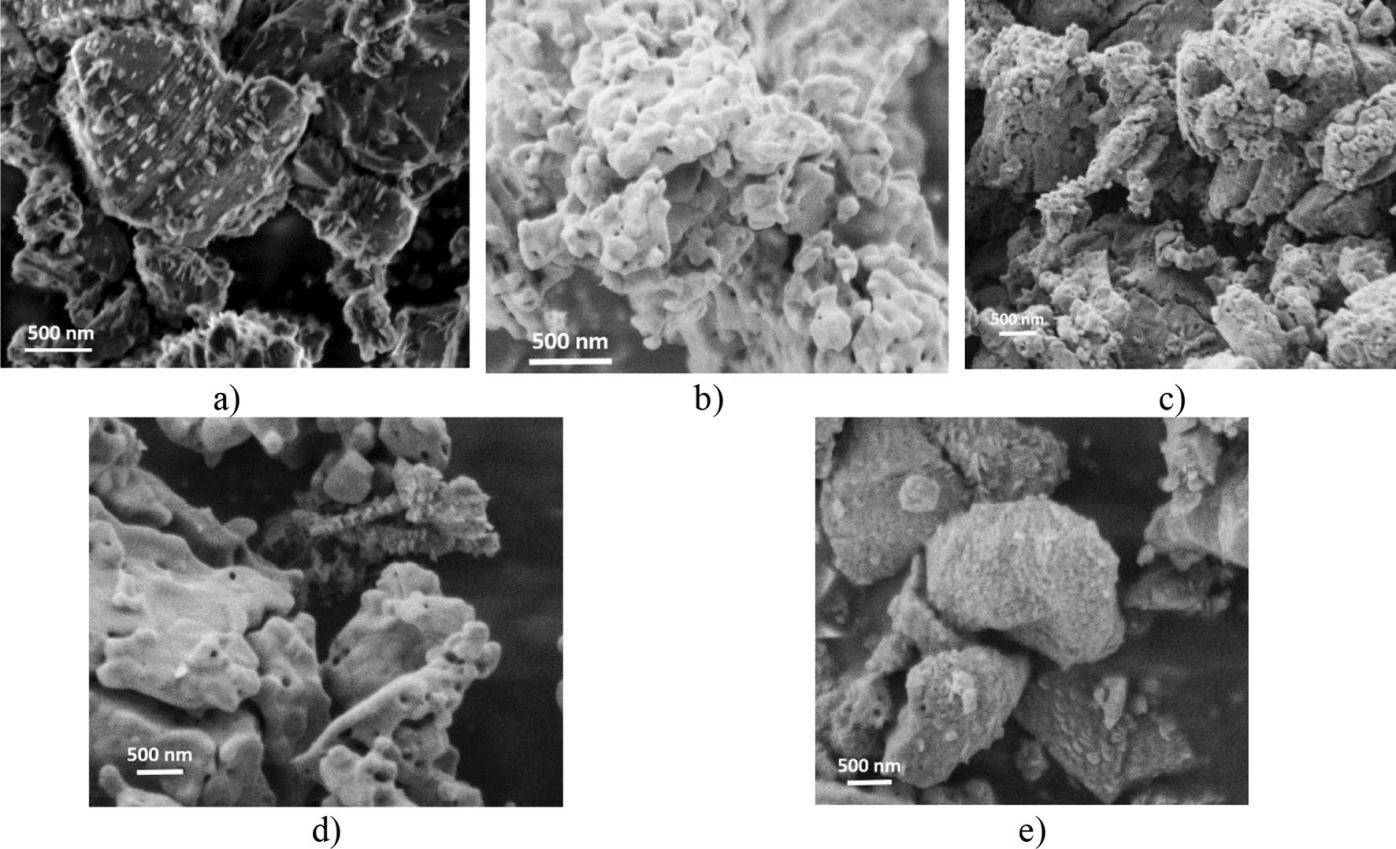
Morphological investigations of oxidized AlN powders. (**a)** reference—without oxidization (O_0), (**b**) 3 h oxidization (O_3), (**c**) 6 h oxidization (O_6), (**d**) 10 h oxidization (O_10) and (**e**) 20 h oxidization (O_20).

**Figure 3:**
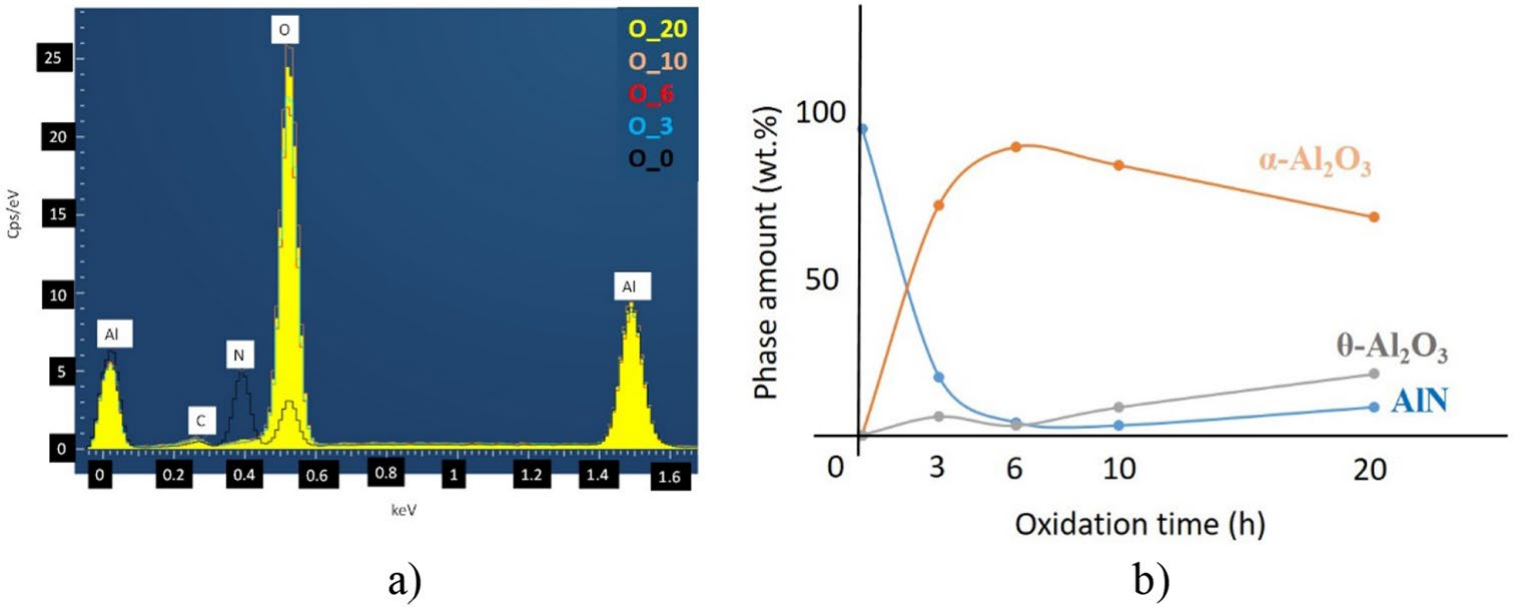
Elemental composition of oxidized AlN. (**a**) Elemental map of powders*, (**b**) quantitative results calculated from XRD. *C is a contamination from carbon tape.

The oxidation behavior of AlN is an important issue. The intermediate unstable phases as δ-Al_2_O_3_, θ-Al_2_O_3_ can be developed during the transformation of AlN to Al_2_O_3_^20^. The studies confirmed that the oxidation mechanism may be described as a reaction process together with a diffusion process. The oxidation process for AlN has been founded in temperatures ranging from 550 to 1100 °C^21–24^. The nearly globular micrometer sized AlN powder has been oxidized at 900 °C from 3 to 20 h in ambient atmosphere (Fig. 2). AlN powder before oxidation showed mainly globular character with average grain size of ~ 1 µm (Fig. 2a). The presence of only the AlN phase has been confirmed by the elemental composition (Fig. 3) analysis. No morphological changes after 3 h oxidization (Fig. 2b) have been observed. The EDS confirmed the presence of oxygen (Fig. 3a) and the quantitative analysis proved the AlN : Al_2_O_3_ ratio to be 19 : 81 wt% (Fig. 3b). Increasing of oxidation time to 6 h slightly increased the grain size of oxidized AlN (Fig. 2c) and the AlN : Al_2_O_3_ ratio is 4 : 96 wt% (Fig. 3).

The increasing of the oxidization time above 10 h caused the formation of pores on the surface of the AlN, indicating the creation of Al_2_O_3_ oxide phase (Fig. 2d,e). The results are in good agreement with the works of Maghsoudipour et al.^11^ and Cao et al.^12^. The particle clustering can also be observed in cases of samples with oxidization time above 6 h.

In our previous study, it has been confirmed simultaneous growth of two different phases of aluminum oxide, α-Al_2_O_3_ and the intermediary θ-Al_2_O_3_ (Fig. 3b). Although the second phase of aluminum oxide can be observed only in the powders after 10 and 20 h of oxidation time^16^. These measurements are in agreement with Tabary et al.^20^.

Sintering of Al_2_O_3_ ceramics by hot isostatic pressing (HIP) has a long history^25^. The advantage of HIP over conventional sintering processes is in obtaining of the very high dense samples. In the case of HIP_10 and HIP_20, the presence of α-Al_2_O_3_ phase has been only observed. It can be explicable by the sintering process in nitrogen and high temperature disintegrated the non-stable θ-Al_2_O_3_ phase^16^. As a post-sintering step pressureless sintering (PS) in hydrogen was applied to as processed HIP samples. Pressureless sintering of AlN-Al_2_O_3_ has been applied to further densify the samples with complex shapes after HIP sintering. The sintered samples (PS_3–PS_20) have been heated at 1800 °C, 0.1 MPa under H_2_ environment to complete the conversion cycle of AlN to Al_2_O_3_.

**Figure 4:**
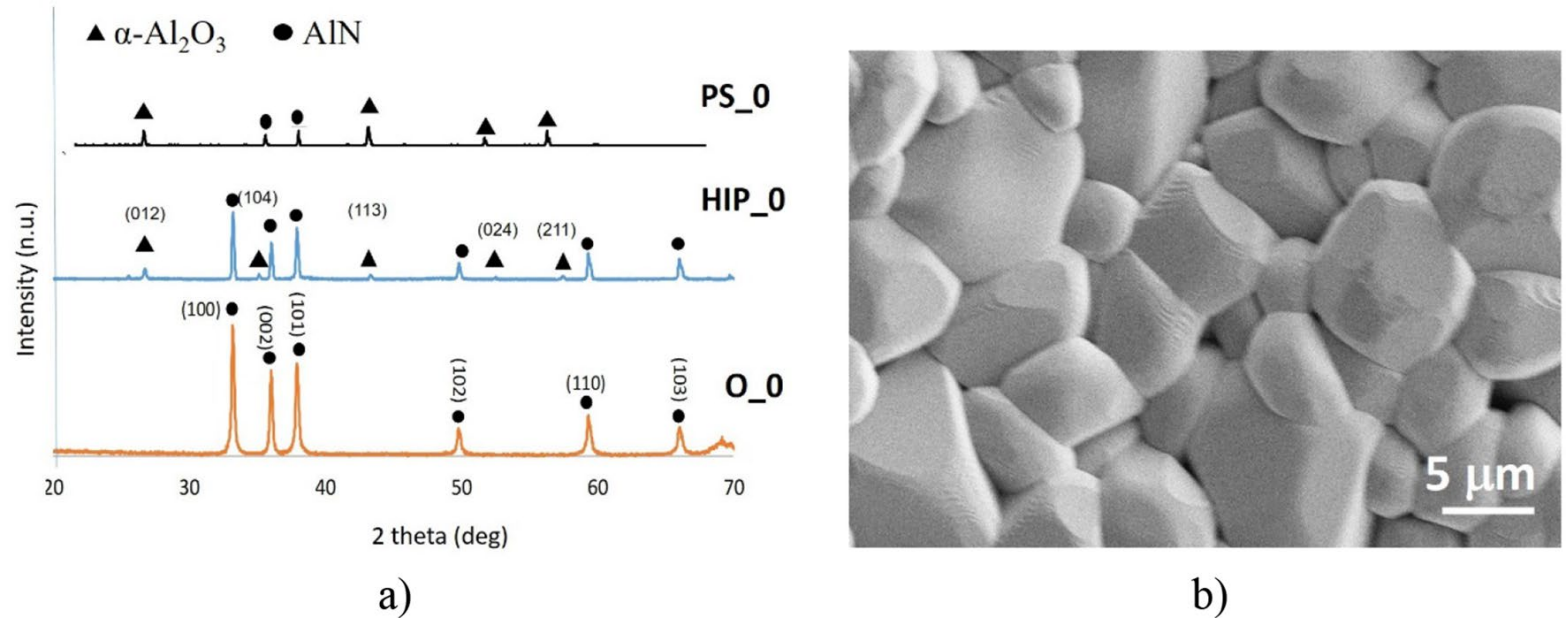
Investigation of AlN reference. (**a**) XRD plots of reference AlN powder (O_0), HIP sintered (HIP_0) and sintered at H_2_ (PS_0), (**b**) SEM image of PS_0.

**Figure 5:**
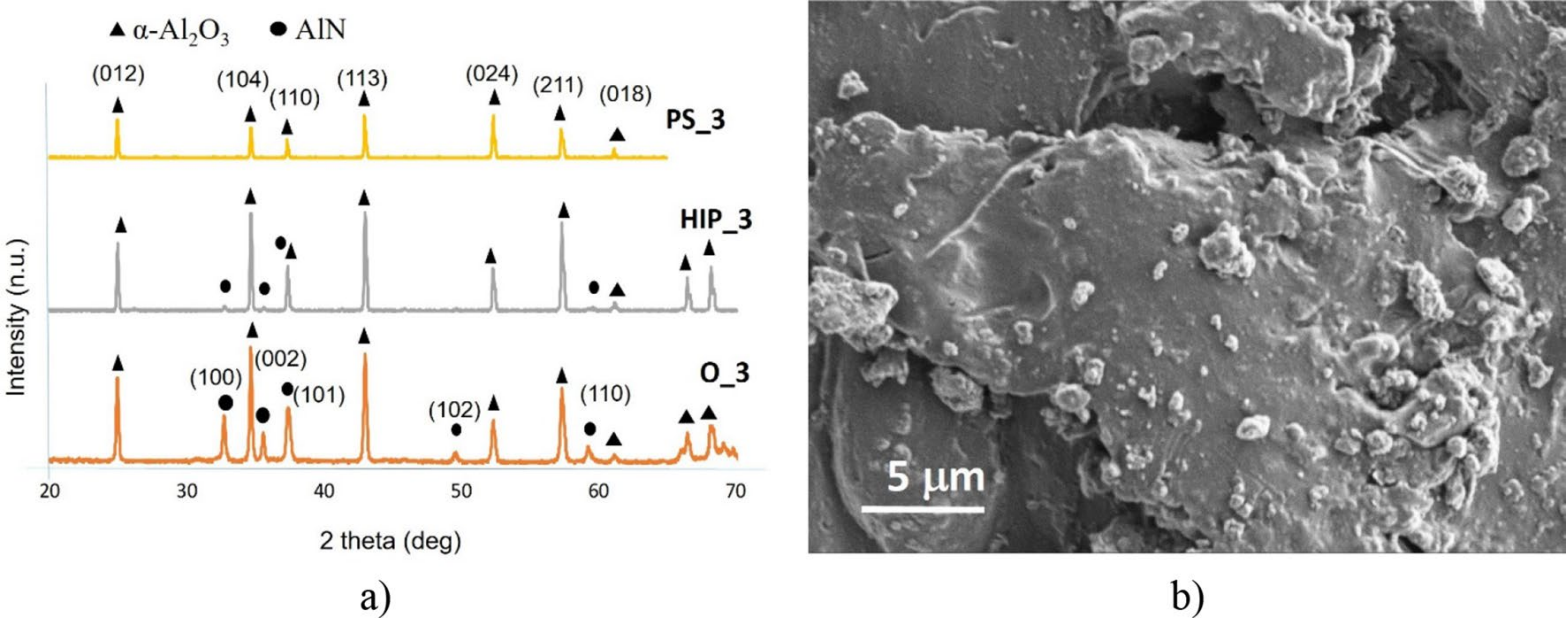
Investigation of oxidized AlN at 3 h. (**a**) XRD plots of oxidized AlN powder (O_3), HIP sintered (HIP_3) and sintered at H_2_ (PS_3), (**b**) SEM image of PS_3.

**Figure 6:**
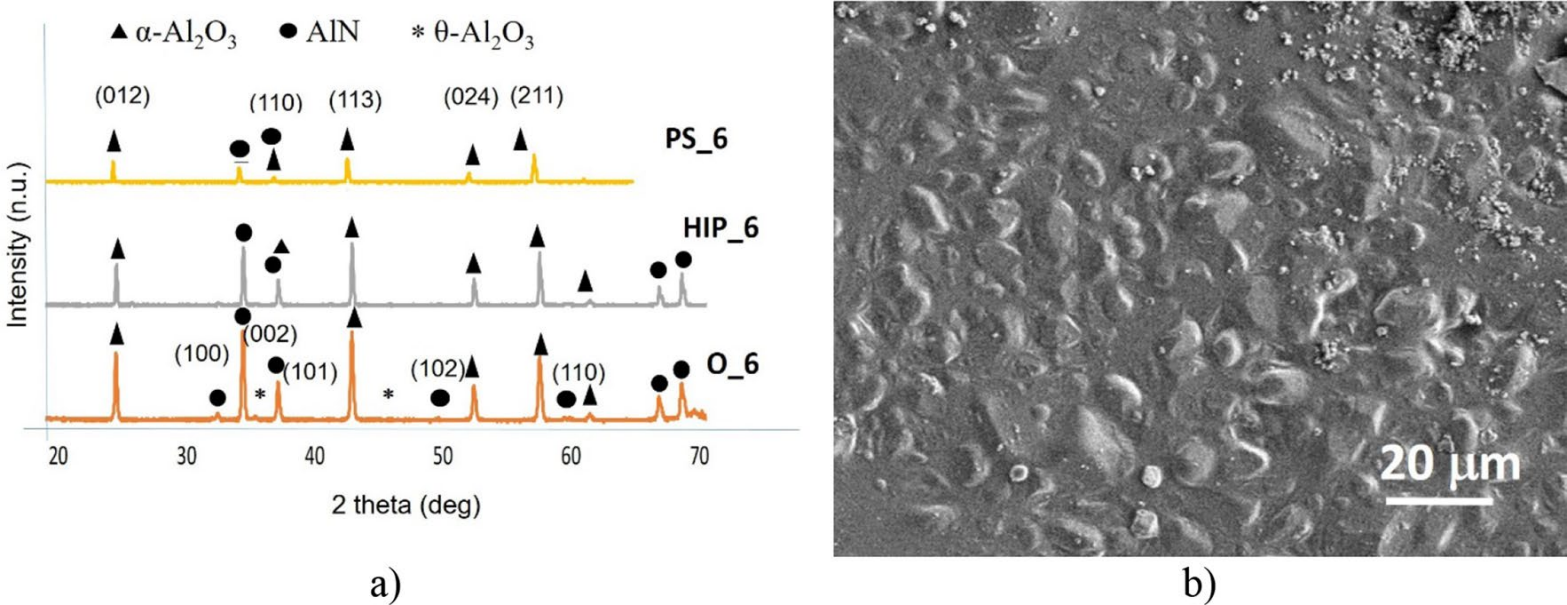
Investigation of oxidized AlN at 6 h. (**a**) XRD plots of oxidized AlN powder (O_6), HIP sintered (HIP_6) and sintered at H_2_ (PS_6), (**b**) SEM image of PS_6.

**Figure 7:**
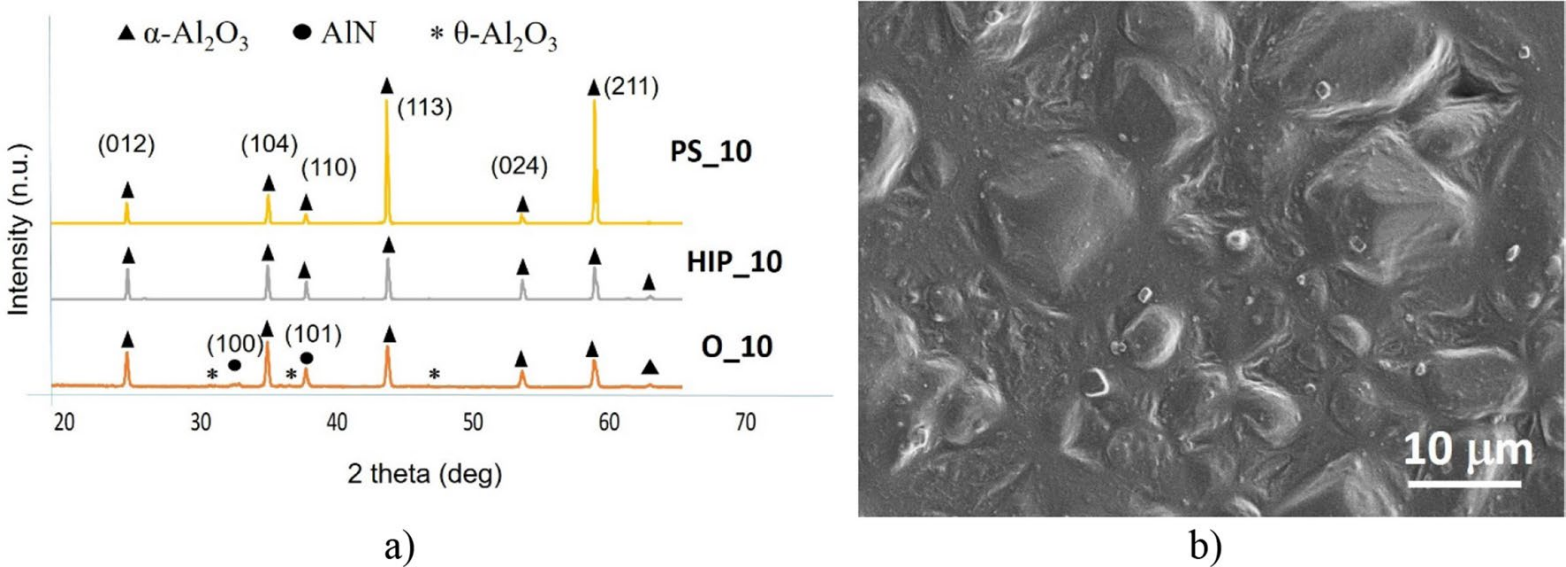
Investigation of oxidized AlN at 10 h. (**a**) XRD plots of oxidized AlN powder (O_10), HIP sintered (HIP_10) and sintered at H_2_ (PS_10), (**b**) SEM image of PS_10.

**Figure 8:**
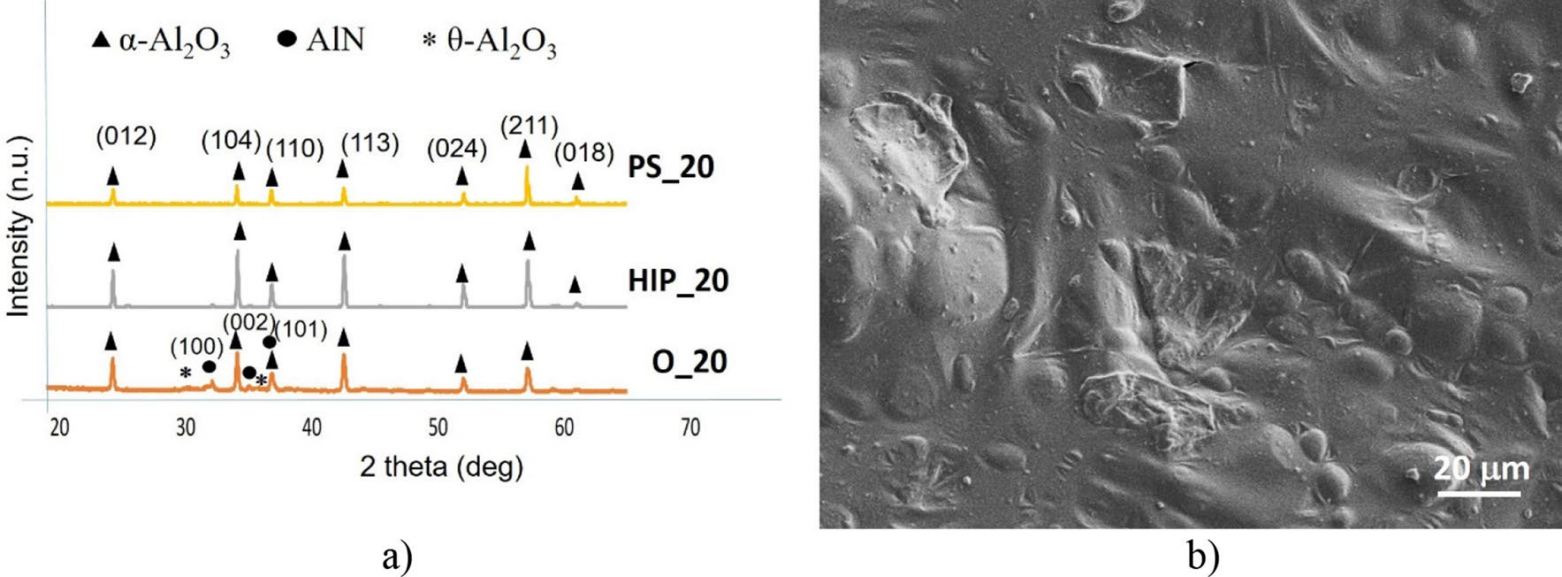
Investigation of oxidized AlN at 20 h. (**a**) XRD plots of oxidized AlN powder (O_20), HIP sintered (HIP_20) and sintered at H_2_ (PS_20), (**b**) SEM image of PS_20.

Comparison of the phase composition of the oxidized powders, HIP sintered and PS sintered samples have been performed by X-ray diffractometry (XRD) (Figs. 4a, 5a, 6a, 7a, 8a). In all samples, hexagonal AlN (JCP2:03–065-1902) and uniform rhombohedral α-Al_2_O_3_ (JCP2:00–010–0173) have been observed as major phases. The oxidation of AlN powders created two new distinct phases of aluminum oxide; major α-Al_2_O_3_ and minor θ-Al_2_O_3_, as it has been shown in our previous work^16^. During the oxidation of AlN, besides α-Al_2_O_3_, formation of various intermediary phases of aluminum oxide, like θ-Al_2_O_3_ and γ-Al_2_O_3_ were observed. However, these oxide phases are unstable and disintegrate at temperatures above 1100 °C^26,27^.

The phase and structural transformation of pure AlN (Fig. 2a) without oxidation has been studied as reference (Fig. 4). The transformation of the part of the AlN to α-Al_2_O_3_ during HIP sintering (Fig. 4b) has been observed. The subsequent PS sintering effected the grain growth from 1 μm to ~ 5 μm (Fig. 4b) at 1800 °C for 10 h. Besson and Abouaf reported that this effect has not been observed only if the pressureless sintering prolonged 100 h at the temperature of 1400 °C^28^.

The comparative phase analysis of the 3 h oxidized AlN and HIP-post PS processes have been presented in Fig. 5. The higher volume of α-Al_2_O_3_ (Fig. 3b) helped the strong phase transformation of remnant AlN to α-Al_2_O_3_ and the sintering in H_2_ finished this process (Fig. 5a). This fact has been confirmed by the XRD results (Fig. 5a). The major reflections corresponding to stable α-Al_2_O_3_ phase occured at 25°, 35°, 43°, 52° and 57° 2θ positions. The PS sintered Al_2_O_3_ has been consisted from the non-uniform morphology (Fig. 5b). The grain size has been still around 5 μm but compared to non-oxidized reference (Fig. 4b), the surface was smoother.

Oxidation above 6 h induced the presence of intermediary θ-Al_2_O_3_ phase. This phase could be topotactically transformed from γ-Al_2_O_3_, which is stable under higher heat-treatment temperatures 800 °C^29^. θ-Al_2_O_3_ is more stable at higher temperatures of ~ 950–1000 °C where kinetic factors play a lesser role^29^. The HIP and PS as well are using comparable higher sintering temperatures, which occured the transformation of the metastable θ-Al_2_O_3_ (Fig. 6a). XRD measurements confirmed mainly α-Al_2_O_3_ with minor AlN phase (Fig. 6a). The morphology of PS sintered sample is shown in Fig. 6b, the sample is characterized by the average grain size ~ 5 μm.

The oxidization above 10 h had the effect on content of AlN. This fact has been supported by the phase and morphological study illustrated in Figs. 7 and 8. In both cases, the presence of metastable θ-Al_2_O_3_ have been proved after oxidation (Figs. 7a, 8a). In all the sintered samples, the composition of Al_2_O_3_ increased as function of oxidation time. The second heating cycle (PS) eliminated all intermediary oxide phases and transformed the substrate into a uniform α-Al_2_O_3_ phase. Therefore, combined sintering (HIP + PS) associated with complete conversion of base AlN to Al_2_O_3_ (Corundum) in the case of longer oxidation time. „The grain size of post-sintered ceramics slightly increased to 10–20 μm in the case of 10 h or 20 h oxidation times.”

**Figure 9:**
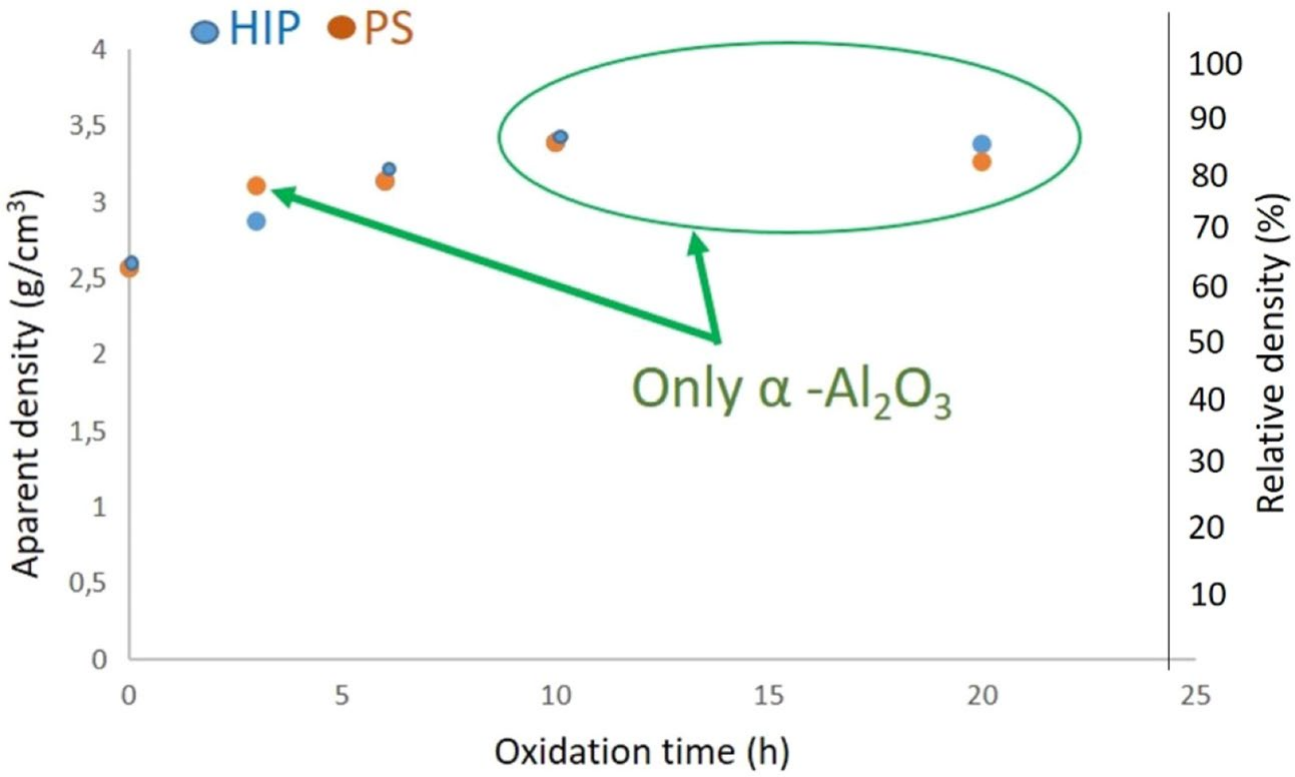
Apparent and relative density measurements of the HIP and PS samples.

The apparent density measurement can help in the valuable information to control the quality of a ceramic with respect to the porosity. The apparent densities of sintered samples (HIP, PS) are shown in Fig. 9. The comparative study of densities of HIP sintered and PS sintered samples showed the similar tendency. The HIP and PS sintered base (reference) AlN exhibited the lowest apparent density (2.57 g/cm^3^, Fig. 9). Increasing of the oxidation time of base AlN powder caused the increasing of density values from 2.87 to 3.38 g/cm^3^ for HIP_3-20 and from 3.11 to 3.27 g/cm^3^ for PS_3-20, respectively. Kim et al. developed the Al_2_O_3_ with additions of 1–25 mol% AlN by the reaction sintering in nitrogen gas at 1600–1800 °C, 20 MPa for 2 h^30^. Sintered Al_2_O_3_ with 1 mol% AlN addition at 1750 °C resulted close to theoretical density of α-Al_2_O_3_ (3.98 g/cm3). They observed for the different compositions of AlN-Al_2_O_3_ that the sintered densities decreased with increasing AlN content^30^.

Apparent density values of HIP and PS sintered Al_2_O_3_ are in agreement with results of group of Kim et al.^30^. The presence only the major α-Al_2_O_3_ predicted the higher densification during sintering process (independently on sintering type) (Fig. 9). AlN phase blocked the fully densification and caused the formation of the bigger grains, porosities and impurities in sintered ceramics (Figs. 4, 5, 6, 7, 8). The highest apparent density 3.39 g/cm^3^ (85% relative density) has been observed in a case of sample oxidized for 10 h.

### Hardness of the sintered Al_2_O_3_

The improvement of the mechanical properties of Al_2_O_3_ can be expected potential ceramics for novel engine or other applications. Hardness is one of the most important mechanical property.

Bocanegra-Bernal et al. obtained hardness of 20.5 ± 0.6 GPa presented a grain size of Al_2_O_3_ ~ 0.62 ± 0.04 µm at the lowest HIP temperature (1300 °C)^31^. Willmann reported the hardness values of 17–19 GPa for grain size of 4.5, 3.2, and 1.8 µm^32^. Xue et al. applied the hot pressing at various temperatures of 1800, 1850, and 1900 °C and produced the AlN-Al_2_O_3_ with hardness between 14 and 16 GPa^33^.

**Figure 10:**
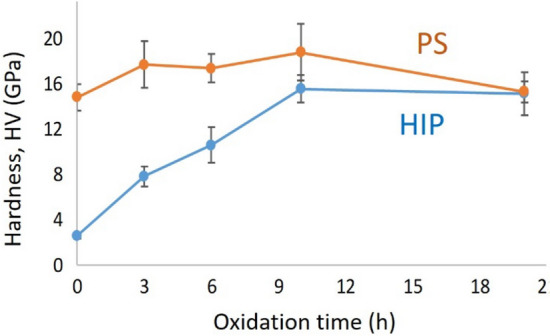
Hardness of HIP and PS sintered Al_2_O_3_ versus oxidation time of AlN base powder.

Hardness values have been characterized as function of oxidation time (Fig. 10). The similar tendency of hardness behavior has been observed for both sintering techniques. The increasing of hardness has been influenced by increasing of oxidization time of base AlN powder, minimal presence of AlN and grain size of α-Al_2_O_3_. In addition, reduction of porosity resulted in closer packing, denser structure and improvement the hardness of sintered samples. The highest hardness values between 17 and 18 GPa have been observed for PS sintered α-Al_2_O_3_ oxidized between 3 and 10 h. These values are comparable with results of other research groups^31–33^.

## Conclusions

Bulk sintered Al_2_O_3_ has been prepared by oxidization of AlN powder and combined sintering process, hot isostatic pressing (HIP) in N_2_ and pressureless sintering (PS) in H_2_ atmosphere. The HIP followed by PS post-sintering of oxidized AlN powder without sintering additives has been successfully developed for the first time. The micrometer sized AlN has been oxidized for 3, 6, 10 and 20 h in ambient atmosphere. The volume of Al_2_O_3_ increased with the increasing of oxidation time of AlN powder. Oxide layer caused porosities and the grains slightly growth. Above 10 h oxidation, “heat-treatment” metastable θ-Al_2_O_3_ phase has been observed. High temperature HIP sintering transformed θ-Al_2_O_3_ and only two major phases α-Al_2_O_3_ and minor AlN have been stabilized. PS post-sintering in 1800 °C for 10 h caused the phase transformation to α-Al_2_O_3_ which had effect on the apparent density and hardness of PS sintered ceramics. The highest apparent densities 3.11–3.39 g/cm^3^ (78–85% relative densities) and highest hardness values (17–18 GPa) have been measured for PS sintered α-Al_2_O_3_ prepared from base powder oxidized between 3 and 10 h.

## Data Availability

All data generated or analysed during this study are included in this published article.
